# Investigation of Enteric Viruses Associated With Runting and Stunting in Day-Old Chicks and Older Broilers in Southwest Nigeria

**DOI:** 10.3389/fvets.2019.00239

**Published:** 2019-07-16

**Authors:** Adebowale I. Adebiyi, Paula L. Tregaskis, Daniel O. Oluwayelu, Victoria J. Smyth

**Affiliations:** ^1^Department of Veterinary Microbiology, University of Ibadan, Ibadan, Nigeria; ^2^Veterinary Sciences Division, Agri-Food and Biosciences Institute, Belfast, United Kingdom

**Keywords:** broilers, enteric virus, runting, stunting, hatchery condemnations, chicken astrovirus

## Abstract

Enteric viruses are known to have significant economic impact on poultry, especially broiler chicken flocks, because of production losses attributable to poor feed conversion and weight gain. To sustain the Nigerian poultry industry that contributes significantly to the livestock sector of the economy, there is a need to investigate commercial broiler flocks in the country for the presence of enteric viruses causing runting and stunting, growth retardation, and hatchery diseases. Gut contents were collected from 158 day-old and six 14-week old runted/stunted broiler chickens in commercial farms (ten) and hatcheries (six) located in Southwest Nigeria. The samples were examined for the presence of chicken astrovirus (CAstV), avian nephritis virus (ANV), avian rotavirus (AvRV), chicken parvovirus (ChPV), and turkey astroviruses (TAstV-1 and−2) by polymerase chain reaction (PCR) and reverse transcriptase-PCR (RT-PCR) whereas avian reovirus (ARV) and fowl adenovirus (FAdV) by virus isolation (VI), RT-PCR, and PCR. While CAstV was detected in all the birds (100%), sporadic detection of ANV (5%), and ChPV (5%) was observed in day-old and/or older birds. Four isolates were obtained by VI with one isolate being ARV positive and other three FAdV positive by RT-PCR and PCR, respectively. These findings strongly suggest CAstV as a major cause of runting and stunting as well as hatchery condemnations in commercial broilers in Southwest Nigeria, although co-infections with ANV, FAdV, ARV, and ChPV cannot be ruled out. In addition, the possible vertical and horizontal transmissions of these viruses are discussed.

## Introduction

Poultry have gradually assumed a very important role in the economy of many industrialized and developing countries as they are a major source of animal protein worldwide. In addition, high demand for chicken meat, egg production, early marketing age, rapid returns of broiler farms, and high profit margins in the lowest possible time have increased the popularity of poultry farming (1, 2). However, optimum performance of the birds is largely dependent on factors including rearing management, feed quality, overall health as well as conditions that affect proper functioning of the gastrointestinal tract such as enteritis which directly decreases feed absorption resulting in growth retardation, impaired feed efficiency, immunosuppression, and sometimes increased mortality due to secondary infections (3, 4).

Enteric viruses cause severe disease conditions such as runting-stunting syndrome (RSS) and White Chick Syndrome (WCS) considered as sources of huge economic losses in poultry production due to culls of stunted, undersized birds that fail to thrive or are too small to pass through the processing plant, increased susceptibility to other diseases, decreased feed conversion efficiency, and prolonged time to market of affected birds (1, 5, 6). Reports have shown severe economic impacts of these diseases with financial losses to affected hatching egg producers and hatcheries estimated at 105,000 US dollars or 68,000 US dollars per 10,000 hens, respectively (2). These enteric viruses of which several RNA and DNA viruses have been implicated, pre-dominantly affect young birds although they may occur in all age groups of poultry. Further, co-infections of multiple viruses such as chicken astrovirus (CAstV), avian nephritis virus (ANV), chicken parvovirus (ChPV), avian rotavirus (AvRV), avian reovirus (ARV), and fowl adenovirus (FAdV) have been reported in birds affected with RSS or with poor performance (6–9). In addition, chickens without symptoms of enteric disease have tested positive for these viruses (9, 10), indicating that they could serve as asymptomatic carriers or reservoirs shedding the virus via the enteric route and thus representing a potential source of infection to clean birds.

**Figure 1:**
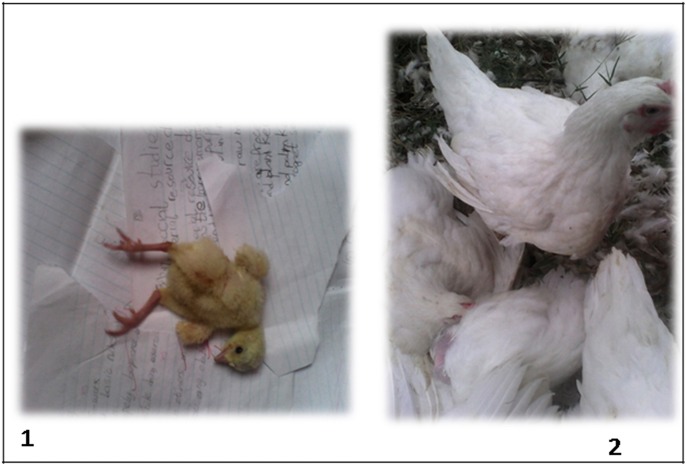
Runted day-old commercial broiler chick showing splayed legs **(1)** and undersized older (14-week-old) commercial broiler chicken **(2)**.

In Nigeria, the agriculture sector accounts for 22.9% of the nation's agricultural gross domestic product (11), with the poultry sub-sector of about 140 million birds (12) contributing significantly to this figure. Moreover, the Nigerian poultry sector is extremely fragmented with the core of the commercial poultry industry located in the southwestern part of the country (13). However, growth of the poultry industry is hampered by reduced flock performance, poor health, high production costs, and economic losses primarily due to the continued menace of infectious diseases. Despite several reports associating enteric viruses with poor poultry performance elsewhere (9, 14, 15), little is known about the connection between these viruses and runting-stunting with growth retardation seen in commercial poultry in Nigeria. Hence, we investigated hatchery-condemned, runted commercial broiler chicks, which are described as undersized at hatch (9, 16) with observed symptoms of weakness, dullness, depression, poor growth, ruffled/wet feathers, and splayed legs and undersized older broiler chickens (Figure 1), for some enteric viruses including CAstV, ANV, ChPV, AvRV, ARV, FAdV, and turkey astrovirus-1 and -2 (TAstV-1 and -2).

## Materials and Methods

This study was carried out in compliance with the National Research Council's guide for animal use and approved by the University of Ibadan Animal Care and Use Research Ethics Committee (UI-ACUREC/18/0116).

### Sample Origin and Processing

Gut contents were obtained from 158 day-old commercial broiler chicks and six 14-week-old adult commercial broiler birds from different commercial hatcheries (six) and/or farms (ten) in Oyo (Ibadan and Ogbomosho), Ogun (Abeokuta), and Osun (Ikirun) States of southwest Nigeria, a region that is generally acclaimed as the poultry hub of the country. Symptoms observed in the sampled day-old chicks were weakness, dullness, depression, poor growth, ruffled/wet feathers, and splayed legs, while the older birds were grossly undersized. These runted day-old birds are usually culled from flocks. The 14-week age of the older commercial broiler birds was found interesting considering the fact that broiler chickens in Nigeria are usually table or market ready by 6–8 weeks of age and this informed the collection of samples (at time of slaughter) from this rare occurrence of very long term rearing by a small-hold farmer. The average flock size on the farms sampled was 2,000 broiler birds with an average number of rejected or culled chicks at 550/production while the commercial hatcheries have an average number of 250,000 per production cycle for commercial broiler chickens with an average number of rejects at 1,540 per production cycle.

The birds were collected on the day of hatch and immediately were euthanized by cervical dislocation and the abdominal cavity dissected open, then a portion of the intestine containing intestinal contents were cut and kept in sample bottles and immediately placed on ice before being transported to the laboratory where they were stored at −70°C until analyzed. Approximately 0.2 g of gut content was removed from the intestinal tract of the chick and added to 1 ml of phosphate buffered–saline (pH 7.2) supplemented with 100,000 μg/ml of Streptomycin, 100,000 units/ml Penicillin and 100,000 μg/ml Amphotericin B in a ribolyser tube containing metal bead and homogenized using a TissueLyser II apparatus (QIAGEN, Hilden, Germany) for 45 s at 30 Hz. The homogenate was transferred to a centrifuge tube, the ribolyser tube rinsed with 1 ml of transport medium and added to the centrifuge tube to give a final 10% (w/v) suspension, followed by centrifugation at 643.4 × g (4°C) for 30 min. The supernatants were stored in pre-labeled vials at −80°C until used for virus isolation. Supernatants from 3 to 5 gut contents were pooled based on flock, location, and source to give a total of 40 pools (Table 1) and used for nucleic acid extraction.

**Table 1:** Quantification of CAstV, ANV, and ChPV by real-time RT-qPCR and qPCR.

**Sample ID**	**Location**	**Source^¢^**	**CAstV log value**	**Sample ID**	**Location**	**Source^¢^**	**CAstV log value**
VF18-p1	Ibadan	H/a	7.04	VF18-p21	Abeokuta	CF/a	6.64
VF18-p2	Ibadan	H/a	6.58	VF18-p22	Abeokuta	CF/a	7.20
VF18-p3	Ibadan	H/b	6.97	VF18-p23	Abeokuta	CF/a	6.99
VF18-p4	Ibadan	SF/a	6.45	VF18-p24	Abeokuta	CF/a	5.99
VF18-p5	Ibadan	SF/b	6.09	VF18-p25	Abeokuta	CF/b	7.10
VF18-p6	Ibadan	SF/a	6.36	VF18-p26	Abeokuta	CF/b	7.19
VF18-p7	Ogbomosho	CF/a	3.80	VF18-p27	Abeokuta	CF/b	5.89
VF18-p8	Ogbomosho	CF/a	3.39	VF18-p28	Ikirun	CF/a	6.98
VF18-p9	Ogbomosho	CF/b	4.12	VF18-p29	Ikirun	CF/a	7.08
VF18-p10	Ogbomosho	CF/c	4.68	VF18-p30	Ikirun	SF/a	7.71
VF18-p11	Ogbomosho	CF/d	2.17	VF18-p31	Ikirun	SF/b	7.85
VF18-p12	Ogbomosho	CF/e	5.90	VF18-p32	Ikirun	SF/c	7.98
VF18-p13	Ogbomosho	CF/e	5.84	VF18-p33	Abeokuta	CF/c	6.91
VF18-p14	Ogbomosho	CF/e	5.20	VF18-p34	Abeokuta	CF/c	7.71
VF18-p15	Ogbomosho	CF/e	5.40	VF18-p35	Abeokuta	CF/d	7.28
VF18-p16	Ogbomosho	CF/f	4.20	VF18-p36	Abeokuta	CF/e	7.40
VF18-p17^μ^	Ogbomosho	CF/f	4.38	VF18-p37	Abeokuta	SF/a	7.19
VF18-p18^μ^^≠^	Ibadan	SF/c	2.22	VF18-p38	Abeokuta	SF/b	6.89
VF18-p19^≠^	Ibadan	SF/c	4.72	VF18-p39	Abeokuta	CF/f	7.65
VF18-p20	Abeokuta	CF/a	7.07	VF18-p40	Abeokuta	CF/f	7.59

*Log values relate to the virus RNA copy numbers stated as logarithmic values (to the base 10)*.

μ *Samples positive for CAstV and ANV*.

≠ *Samples positive for CAstV and ChPV. VF18-p18 and VF18-p19 were from stunted 14-week-old commercial broilers while others were from day-old runted commercial broiler chicks*.

¢ *Upper case letters denote different poultry establishments (H, hatchery; SF, smallholder farm; CF, commercial farm). Lower case letters indicate different farms/flocks*.

### Cell Culture Viral Isolation for Cytopathic Effects (All Samples/Not Pooled)

Viruses were isolated via *in vitro* infection of primary chicken embryo liver (CEL) cell culture. Briefly, 1 ml suspensions of primary CEL cells from 14 to 16-day-old specific-pathogen-free chicken embryos were seeded into 14 ml glass tissue culture roller tubes, maintained in 1 ml Medium 199 supplemented with 10% fetal calf serum (FCS) and incubated at 37°C with 5% CO_2_ overnight to form a monolayer. The supernatant was removed aseptically and 1 ml of Medium 199 containing 2% FCS was added to the tubes. The supernatants of gut contents lysates were filtered through a 0.22 μm filter, and 100 μl was added to the medium in each tube. After 6 days incubation, the monolayers were examined for cytopathic effect (CPE) and then freeze-thawed three times. If no CPE was visible, a second passage was performed as described above inoculating 100 μl of the first passage cell suspension onto a fresh CEL monolayer for up to three passages.

#### RNA and DNA Extraction From Cell Culture Samples With CPE

Viral RNA and DNA were extracted from the frozen and thawed cultures using the QIAmp Viral RNA Mini Kit (QIAGEN, Manchester, UK) according to manufacturer's instructions and stored at −80°C until tested for ARV and FAdV by RT-PCR and PCR, respectively. It was previously determined that the QIAmp Viral RNA Mini Kit co-purifies DNA as well as RNA (9).

### Molecular Detection of Enteric Viruses

The names and sequences of primers used for RT-PCR and PCR reactions in this study are shown in Table 2.

**Table 2:** Primers used in the present study and their nucleotide sequences.

**Primer**	**Nucleotide sequence (5^′^−3^′^)**	**Target gene**	**References**
CAstV F	GCYGCTGCTGAAGAWATA CAG	Polymerase	(7)
CAstV R	CATCCCTCTACCAGATTTTCT GAA A		
Probe	6-FAM-CAG AAG TCG GGC CC-MGB		
ANV F	GTA AAC CAC TGG YTG GCT GAC T	Polymerase	(7)
ANV R	TAC TCG CCG TGG CCT CG		
Probe	6-FAM-CAG CAA CTG ACT TTC-MGB		
CAstV (pre cap) F	TAG AGG GAT GGA CCG AAA TAT AGC AGC	ORF 2 (capsid)	(17)
CAstV (post-cap) R	TGC AGC TGT ACC CTC GAT CCTA		
FAdV Hex(A) F	CAA RTT CAG RCA GAC GGT	Hexon	(18)
FAdV Hex(B) R	TAG TGA TGM CGS GAC ATC AT		
ARV (Reo) P1F	AGT ATT TGT GAG TAC GAT TG	Sigma C	(19)
ARV (Reo) P4R	GGC GCC ACA CCT TAG GT		
AvRV (ROT) F	GGG CGT GCG GAA AGA TGG AGA AC	NSP4	(20)
AvRV (ROT) R	GGG GTT GGG GTA CCA GGG ATT AA		
TAstV-1 F	AGCTYATGMGGTTCTTTCTTCTYG	Polymerase	(20)
TAstV-1 R	GATGGTGGGTAGCCTATTGTGTTC		
TAstV-2 F	TGGACCGACCCRRTTTTYACCA	Polymerase	(20)
TAstV-2 R	GGCCCGACYTCAGGMAGTTGT		

#### Nucleic Acid Extraction From the Pooled Samples

Automated extraction of nucleic acids was performed on the MagNA Pure 96 robotic workstation (Roche, Burgess Hill, UK), according to manufacturer's instructions. Positive and negative extraction controls consisting of identified virus stock and phosphate buffered saline (PBS) pH 7.2, respectively, were included with each batch of test sample supernatants extracted. The nucleic acids were stored at −80°C until tested for CAstV, ANV, ChPV, AvRV, TAstV-1, and -2 by real-time and conventional RT-PCR or PCR as appropriate.

#### Real-Time RT-PCR (CAstV and ANV)

Real-time RT-PCR (RT-qPCR) reactions were set up in triplicate per sample with a total volume of 20 μl per replicate reaction. Each reaction comprised 10 μl AgPath-ID^TM^ One-Step RT-PCR 2× buffer (ThermoFisher Scientific, MA, USA), 0.8 μl AgPath-ID^TM^ One-Step RT-PCR enzyme, 1 μl of primer- probe mix (containing primers to a final concentration of 400 nM and probe to a final concentration of 120 nM), 2 μl sample or positive control RNA and nuclease-free distilled water (dH_2_O) to 20 μl. The 2 μl of sample RNA was replaced in the PCR negative control by 2 μl dH_2_O or 2 μl of negative extraction control and this holds true for all subsequent PCRs. RT-qPCR for CAstV and ANV were performed as previously described (7). The reactions were carried out in a 7,500 Real-Time PCR System (ThermoFisher Scientific) starting with a reverse transcription step at 45°C for 10 min, then an initial denaturation step at 95°C for 10 min, followed by 40 cycles of denaturation at 95°C for 15 s, and then primer annealing and template amplification at 60°C for 45 s, while fluorescence readings were taken during the amplification stage. During post-PCR analysis, cycle thresholds were set while the reactions were in true exponential phase prior to the linear phase. The log (base 10) values of genome copies were calculated as described by Smyth et al. (7).

#### Real-Time PCR (Chicken and Turkey Parvovirus)

Primers and hydrolysis probe that amplify all known strains of chicken and turkey parvovirus were designed in Agri-Food and Biosciences Institute (AFBI), Belfast, UK. Each reaction comprised 10 μl Brilliant III UltraFast qPCR Master Mix (Agilent Technologies, CA, USA), 0.3 μl Rox dye, 1.5 μl of primer-probe mix (primers to a final concentration of 400 nM and probe to a final concentration of 120 nM), 2 μl sample or positive control DNA and nuclease-free dH_2_O to 20 μl. Real-time PCR for ChPV was performed under fast cycling conditions in a 7500 Fast Real-Time PCR System (Thermo Fisher Scientific) comprising 95°C for 3 min, followed by 40 cycles of denaturation at 95°C for 12 s, and then primer annealing and template amplification at 60°C for 30 s including data capture.

#### Conventional RT-PCR and PCR (ARV, AvRV, TAstV, CAstV, and FAdV)

Conventional RT-PCR was carried out for ARV on cell culture isolates using the REO P1F & P4R primers described in Kant et al. (19), which amplify part of the open reading frame (ORF) of the S1 segment encoding the sigma C protein while ROT forward and reverse primers that amplify non-structural protein 4 (NSP4) of group A AvRVs (20) were applied to RNAs extracted from gut contents. Also, TAstV 1F and 1R, and 2F and 2R primers that target the polymerase gene (ORF 1B) of TAstV-1 and TAstV-2, respectively (20), were used on RNAs extracted from gut contents. A reaction mix comprising 12.5 μl AffinityScript^TM^ One-Step RT-PCR 2 × buffer (Agilent Technologies), 0.5 μl AffinityScript^TM^ One-Step RT-PCR enzyme (Agilent Technologies), 2.5 μl RNA template, primers to a final concentration of 400 nM and diethyl pyrocarbonate (DEPC) H_2_O to a final volume of 25 μl was used. The thermal cycling conditions for ARV and AvRV were one cycle of reverse transcription (45°C for 30 min), one cycle of initial denaturation (94°C for 2 min), and 40 cycles of amplification (94°C for 15 s, 58°C for 30 s, 68°C for 45 s, and 68°C for 7 min). The cycling conditions for TAstV-1 and TAstV-2 were the same as above except for the annealing temperature (55°C for 30 s).

In addition, 32 pools of sample RNAs that contained substantial quantities of CAstV by RT-qPCR were amplified by conventional RT-PCR of the ORF 2 (capsid) gene as described previously (17).

Polymerase chain reaction targeting the hexon gene of FAdV (18) was performed with adenovirus Hex A and B primers using Taq PCR Master Mix kit (QIAGEN). Briefly, 25 μl reaction mixes contained primers to a final concentration of 400 nM, 2.5 μl DNA template, Taq DNA polymerase and DEPC H_2_O. The thermal cycling conditions were one cycle of denaturation (95°C for 15 min) followed by 35 cycles of amplification (94°C for 30 s, 62°C for 1 min, 72°C for 1 min, and 72°C for 7 min).

#### Analysis of RT-PCR and PCR Products

The RT-PCR and PCR products were analyzed by electrophoretic diffusion in 1.5% agarose gels submerged in 1X Tris-acetate ethylene diamine tetra-acetic acid (TAE) buffer. The size of DNA fragments was estimated by comparison with a 1 kb Plus DNA Ladder (Thermo Fisher Scientific) under UV trans-illumination.

### Sequence and Phylogenetic Analyses

All the 32 CAstV RT-PCR products with high RNA viral copies as well as those of ANV (*n* = 1), ChPV (*n* = 1), and ARV (*n* = 1) were purified using PureLink extraction kit (Thermo Fisher Scientific). The products were sequenced commercially. Analysis of sequencing data and multiple alignments were performed using the ClustalW method integrated in Geneious v8.0 software (Biomatters, Auckland, New Zealand).

Phylogenetic analysis was carried out using the neighbor-joining (21) algorithm with 1,000 bootstrap replicates in the Molecular Evolutionary Genetics Analysis (MEGA) X software (22). The evolutionary distances were computed using the Maximum Composite Likelihood method (23) and are in the units of the number of base substitutions per site. The sequences of all the reference strains used were obtained from the GenBank database (Tables 3A–D).

**Table 3A:** CAstV reference strains for phylogenetic analysis.

	**Identity**	**Location**	**Accession number**
1	VF06-1/4	United Kingdom	JN582309.1
2	VF06-7/5	United Kingdom	JN582310.1
3	CAstV 11522	United States	JN582305.1
4	CAstV 11672 Bi	United Kingdom	JN582327.1
5	VF11-66B WC	Finland	Unpublished (from AFBI)
6	CAstV WC	Canada	KY635970.1
7	PRDC/533 south zone	India	JX945859.1
8	PRDC/576 north zone	India	JX945883.1
9	PRDC/574 north zone	India	JX945862.1
10	CAstV/01/17/HR	India	MF405736.1
11	Astrovirus isolate 301-4	Italy	JQ307839.1

**Table 3B:** ARV reference strains for phylogenetic analysis.

	**Identity**	**Location**	**Accession number**
1	Strain 284-V-06 NS protein gene	Hungary	KX398238.1
2	16821-M-06 NS protein gene	Hungary	KX398308.1
3	Isolate Reo/BC/Broiler/16-0753A/16 sigma C (S1) gene	Canada	MG822677.1
4	Isolate Reo/BC/Broiler/16-0753B/16 sigma C (S1) gene	Canada	MG822676.1
5	Isolate Reo/BC/Broiler/16-0711/16 sigma C (S1) gene	Canada	MG822679.1
6	Strain T1781 segment S1	Hungary	KC865792.1
7	Isolate Reo/PA/Broiler/07634/14 sigma C gene	USA	KR856992.1
8	Isolate 100192 sigma C (S1) gene	USA	KJ879700.1
9	Isolate 99848 sigma C (S1) gene	USA	KJ879690.1
10	Isolate 99847 sigma C (S1) gene	USA	KJ879689.1
11	Isolate 97362 sigma C (S1) gene	USA	KJ879648.1
12	Isolate 99477 sigma C (S1) gene	USA	KJ879653.1
13	Isolate 95403 sigma C (S1) gene	USA	KJ803969.1
14	Isolate Reo/PA/Broiler/22790/11 sigma C gene	USA	KP727787.1
15	Isolate Reo/PA/Layer/03422/14 sigma C gene	USA	KP727788.1

**Table 3C:** ChPV reference strains for phylogenetic analysis.

	**Identity**	**Location**	**Accession number**
1	Chicken parvovirus Strain ChPV/Poland/G090	Poland	JQ178302.1
2	Chicken parvovirus isolate ChPV CAN-5	Canada	JF267314.1
3	Turkey parvovirus strain TuPV/Poland/G048	Poland	JQ178321.1
4	Turkey parvovirus strain Tu1/VA/00	USA	JX207118.1
5	Turkey parvovirus strain TuPV/Poland/G006	Poland	J178317.1
6	Chicken parvovirus isolate USP 238-1	Brazil	MH176307.1
7	Chicken parvovirus isolate ChPV CAN-41	Canada	JF267318.1
8	Chicken parvovirus Ch1515/2007/HUN	Hungary	HM208288.1
9	Turkey parvovirus isolate CRO-844	Croatia	JX114938.1
10	Turkey parvovirus strain Tu3/PA/09	USA	JX207131.1
11	Turkey parvovirus isolate CRO-876	Croatia	JX114940.1
12	Chicken parvovirus isolate CAN-50	Canada	JF267322.1
13	Turkey parvovirus Tu762/2009/HUN	Hungary	HM208287.1
14	Turkey parvovirus isolate TuPV/LT521	USA	KU569262.1

**Table 3D:** ANV reference strains for phylogenetic analysis.

	**Identity**	**Location**	**Accession number**
1	ANV isolate GA-CK-SEP AN-368-2005	USA	HQ188694.1
2	ANV isolate 3	Iran	KC811068.1
3	ANV isolate GA-CK-SEP AN-458-2005	USA	HQ1880699.1
4	ANV isolate DE-CK-SEP AN-811-2005	USA	HQ1880693.1
5	ANV strain 45-4	Brazil	KU711059.1
6	ANV strain 46-1	Brazil	KU711065.1
7	ANV strain 46-4	Brazil	KU711064.1
8	ANV strain 46-2	Brazil	KU711063.1

## Results

### Virus Isolation

**Figure 2:**
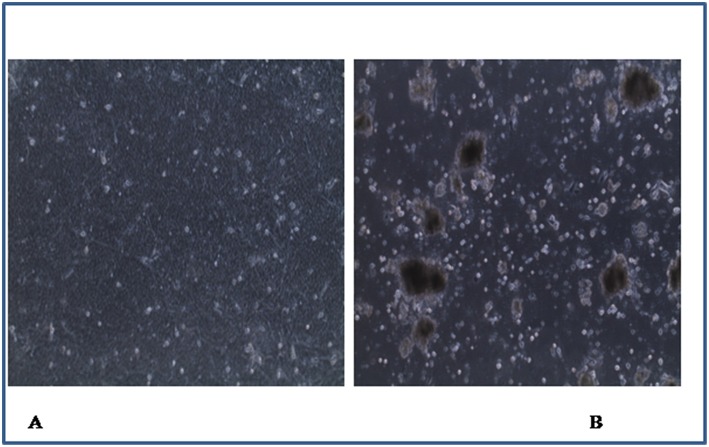
Chicken embryo liver cells ( ×40): **(A)** control with uninfected monolayer cells, **(B)** infected cells showing cell death and detachment.

Four of the 164 samples induced CPE characterized by cell death and detachment (Figure 2) at different passage levels in infected CEL cell cultures (Table 4).

**Table 4:** Virus Isolation (VI) and typing by PCR and sequencing.

	**Positive samples**		
**Sample**	**VI passage levels**	**PCR/Sequencing**	
		**FAdV**	**ARV**
VF18-B24	1p	Serotype 4	–
VF18-B26	3p	Serotype 5	–
VF18-B75	1p	–	GEL 13b98 strain
VF18-B109	1p	Serotype 4	–

*VF18-B75 was from stunted 14-week-old broilers while others were from day-old runted chicks. FAdV, fowl adenovirus (data on sequencing not shown); ARV, avian reovirus*.

### Real-Time RT-PCR and PCR

The results of real-time RT-qPCR and qPCR showed that all the 40 tested pools were positive for CAstV with RNA log_10_ values ranging from 2.17 to 7.98 for day-old chicks and 2.22 to 4.72 for the 14-week-old birds (Table 1).

Majority (25/40) of the pools were considered to have high (>6.0) RNA log_10_ values, followed by 11 pools with medium (4.0–6.0) RNA log_10_ values and a few (4/40) with low (<4.0) RNA log_10_ values as described by Smyth et al. (7). In addition, pool VF18-p17 (from day-old chicks) was positive for CAstV and ANV (RNA log_10_ value of 2.8); VF18-p19 (from 14-week-old broilers) was positive for CAstV and ChPV (DNA log_10_ value of 2.59); meanwhile pool VF18-p18 was positive for CAstV, ANV (RNA log_10_ value of 4.88), and ChPV (DNA log_10_ value of 3.23).

### Conventional RT-PCR and PCR

Conventional assays carried out on the four cell culture isolates detected ARV and FAdV in one and three samples, respectively (Table 4). In addition, all the pooled samples tested negative for AvRV, TAstV -1, and -2 while the 32 RT-qPCR-positive pools tested were all positive for CAstV capsid gene. A summary of detected enteric viruses in commercial broilers with runting and stunting problems in the present study is shown in Table 1.

### Sequence and Phylogenetic Analyses

**Figure 3:**
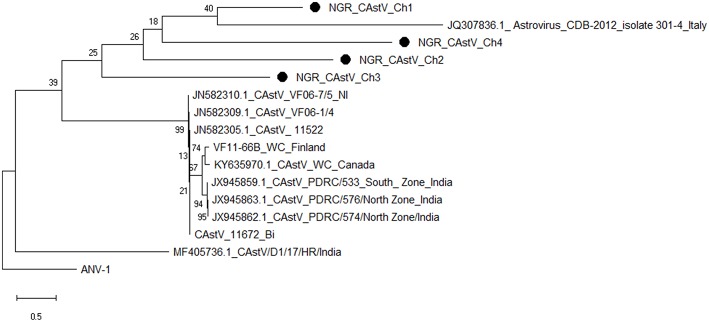
Phylogenetic tree based on analysis of partial nucleotide sequences of the chicken astrovirus (CAstV) open reading frame (ORF) 2 (capsid) gene. The Nigerian CAstVs (NGR-CAstV-Ch) sequenced in this study are marked with a solid black circle; other CAstV sequences are reference strains obtained from the GenBank database (Table 3A). The phylogenetic tree was generated using MEGAX software employing the neighbor-joining method and a 1,000 bootstrap analysis. The scale bar is 0.5.

**Figure 4:**
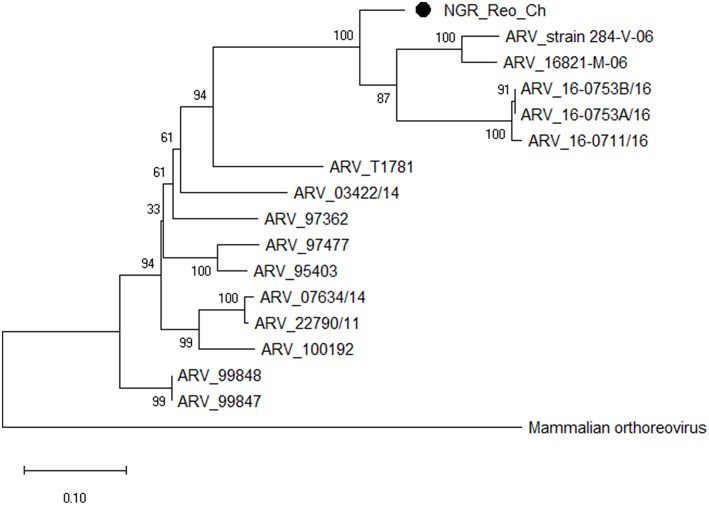
Phylogenetic tree based on analysis of partial nucleotide sequences of the avian reovirus (ARV) sigma C protein gene. The Nigerian avian reovirus isolate (NGR-Reo-Ch) sequenced in this study is marked with a solid black circle; other ARV sequences are reference strains obtained from the GenBank database (Table 3B). The phylogenetic tree was generated using MEGA X software employing the neighbor-joining method and a 1,000 bootstrap analysis. The scale bar is 0.1.

**Figure 5:**
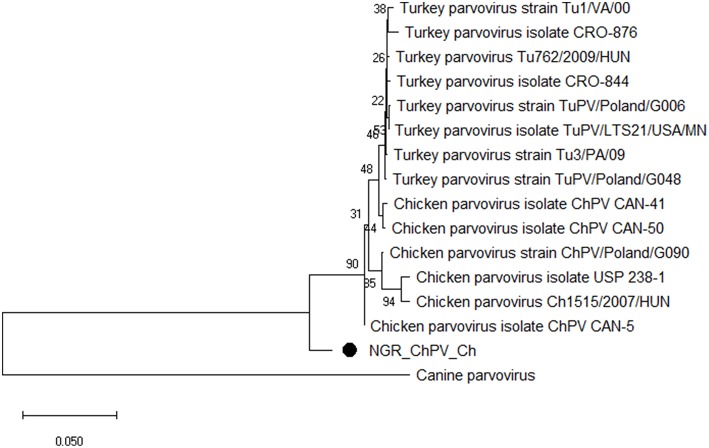
Phylogenetic tree based on analysis of partial nucleotide sequences of the chicken and turkey parvoviruses non-structural protein gene. The Nigerian chicken parvovirus (NGR-ChPV-Ch) sequenced in this study is marked with a solid black circle; other parvovirus sequences are reference strains obtained from the GenBank database (Table 3C). The phylogenetic tree was generated using MEGAX software employing the neighbor-joining method and a 1,000 bootstrap analysis. The scale bar is 0.05.

**Figure 6:**
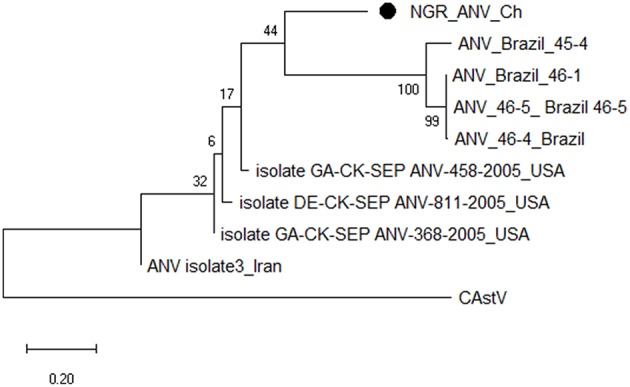
Phylogenetic tree based on analysis of partial nucleotide sequences of the avian nephritis virus (ANV) open reading frame (ORF) 2 (capsid) gene. The Nigerian ANV (NGR-ANV-Ch) sequenced in this study is marked with a solid black circle; other ANV sequences are reference strains obtained from the GenBank database (Table 3D). The phylogenetic tree was generated using MEGAX software employing the neighbor-joining method and a 1,000 bootstrap analysis. The scale bar is 0.2.

Multiple sequence alignment of obtained CAstV nucleotide sequences showed they were 98–100% homologous with group Bi CAstVs by capsid gene analysis (17). Based on analysis of the sigma C protein, the Nigerian ARV strain (NGR-Reo-Ch) shared 85% similarity with isolate Reo/BC/Broiler/16-0753A/16 which is classified in cluster 3 and was obtained between 2012 and 2017 from cases of viral arthritis in Western Canada (24). The Nigerian ChPV identified (NGR-ChPV-Ch) was 96% homologous with enteric parvovirus strain G090/2011 isolated from commercial turkey and chicken flocks in Poland (25). The only ANV sequence obtained in this study (NGR-ANV-Ch) shared 80% similarity by the ORF2 (capsid) gene with ANV-2 from chickens in Japan and United Kingdom (26, 27). All nucleotide sequences obtained in this study were submitted to GenBank under accession numbers MK509014 (NGR_CAstV_Ch1), MK509015 (NGR_CAstV_Ch2), MK518374 (NGR_CAstV_Ch3), and MK518375 (NGR_CAstV_Ch4) for Nigerian CAstV; MN026334 (NGR_ARV_Ch) for Nigerian ARV; MN026333 (NGR_ChPV_Ch) for Nigerian ChPV and MN026335 (NGR_ANV_Ch) for Nigerian ANV. The phylogenetic trees for CAstV, ARV, ChPV, and ANV are shown in Figures 3–6.

## Discussion

Enteric viruses in broiler chicken flocks are considered to have significant economic impact because of production losses due to poor feed conversions and weight gain. Earlier studies in Nigeria reported the detection of CAstV and ANV genome, and CAstV antibodies in indigenous chickens (28, 29). In this study, we investigated commercial day-old and adult broiler chickens obtained from hatcheries and farms in southwest Nigeria for enteric viruses associated with runting and stunting problems.

The detection of CAstV in all the birds with majority (62.5%, 25/40) of them having high levels of viral RNA as well as sporadic detection of FAdV and ANV in day-old chicks, and ANV, ChPV, and ARV in the older birds shows that enteric viruses may infect all age groups of poultry. This is consistent with the report of Nunez et al. (1) who also detected CAstV and ANV in intestinal samples of broilers with RSS. Although several studies worldwide (1, 7, 8, 30) have detected these enteric viruses in malabsorption diseases such as RSS in chickens, our findings support the proposition that CAstV is the major aetiological agent of RSS in poultry (31, 32) since not only were all the 40 sample pools positive for CAstV alone, but the strains of the virus detected in 32 of the samples tested were virtually identical. This strongly suggests that this particular strain is the cause of the runting-stunting and hatchery condemnations seen in the sampled birds. However, it is contrary to the report of Smyth et al. (17) that there are usually many different strains of CAstV in circulation often with low shared genetic identity. The presence of the same CAstV strain in older, stunted birds may indicate that it has the potential to persist in flocks and could perpetuate stunting, especially if it was contracted early. It is less likely to have been contracted as a later infection since chickens rapidly develop resistance to the effects of CAstV and no strains have yet been identified that are known to cause problems in older birds (6).

Recent studies have revealed that CAstV infections are common in broiler chickens and have strong associations with diseases of young birds and hatchery disease (1, 6, 33). In addition, CAstV has been reported to be either transmitted horizontally by fecal-oral route or vertically via naive in-lay parent birds (1, 7). In this study, CAstV was the only virus detected in all of the tested gut contents from runted/stunted day-old and 14-week-old broiler chickens. This detection of the virus in day-old broilers suggests vertical transmission of CAstV and implies that the parent stock transferred high titers of virus to the chicks. Likewise, the detection of the virus in adult broilers may indicate persistent infection that has not been cleared up completely. The high level of resistance of CAstV to commonly used disinfectants (34) may have contributed to this persistence in the older birds via possible sustained contamination of the environment. We speculate that the retarded growth of the older birds was probably due to early infection by this virus causing stunting and allowing it to persist in these birds at moderate levels in hatcheries and farms across southwest Nigeria.

Avian nephritis virus, like CAstV, has been implicated in growth depression including uneven growth and RSS, with reports of simultaneous identification of both viruses in chickens with RSS (5, 7). Although we detected ANV and CAstV in day-old and adult broilers in this study, the frequency of detection of ANV was much less than that of CAstV, especially in day-old chicks. This contrasts with the detection of greater number of ANV than CAstV-infected samples elsewhere (7, 35). Further, ANV is a cause of baby chick nephropathy and is expected to be found in sick hatchlings, but surprisingly CAstV, rather than ANV, was detected in all the runted day-old chicks in this study. This has parallels with the White Chicks' hatchery disease in which vertically transmitted CAstV strains have been implicated as causative agent (6).

Fast-growing broilers are the most susceptible to ChPV which has also been identified as a cause of RSS and enteritis in chickens. Moreover, it has been reported that in natural or experimental infections, this virus causes growth retardation, poor feathering, and bone disorders in broiler chickens with most infections occurring within the first week of age (36, 37). However, in this study, there was no detection of ChPV in the day-old broiler chicks. Conversely, the two pooled samples (VF18-p18 and VF18-p19) from the 14-week-old birds were positive for the virus. This may be due to the fact that infected older birds shed high concentrations of the virus in their feces, thus contributing to fast and efficient horizontal bird-to-bird transmission of the infection (37).

Chicken astrovirus, ANV, ChPV, FAdV, and ARV are important pathogens responsible for poor growth performance and silent losses in the poultry industry. These viruses have been detected in chicken flocks with growth retardation, bad feathering and enteritis worldwide (8, 30, 38). Therefore, their detection in day-old broiler chicks in this study corroborates earlier reports that broiler birds are most susceptible to viral infections during the early post-hatching period (30, 38), and further confirms that these viruses are vertically transmitted. However, the detection of CAstV, ANV, and ChPV in older birds is an indication of persistent infection and horizontal transmission.

We conclude that since CAstV is the only virus detected in all samples from day-old chicks and 14-week-old birds, and because it is present at high titres in most of the hatchery-condemned chicks, it is most likely the major cause of RSS in broilers in southwest Nigeria; ANV, ChPV, FAdV, and ARV were only detected infrequently. Our findings underscore the economic significance and impact of these enteric viruses, especially CAstV on profitable broiler production in the study area since these birds are culled thus reducing financial viability of farmers. In order to achieve effective prevention and control of these viruses, we therefore, advocate strict enforcement of biosecurity measures as well as vaccination of broiler breeder flocks, as appropriate.

## Data Availability

All data generated or analyzed during this study are included in this published article. The raw data are available from the authors upon request.

## Ethics Statement

This study was carried out in compliance with the National Research Council's guide for animal use and approved by the University of Ibadan Animal Care and Use Research Ethics Committee (UI-ACUREC/18/0116).

## Author Contributions

AA, DO, and VS contributed to the conception and design of the study. AA, PT, DO, and VS were involved in the acquisition, analysis, and interpretation of data. AA wrote the first draft of the manuscript. All authors contributed to manuscript revision, read, and approved the submitted version.

### Conflict of Interest Statement

The authors declare that the research was conducted in the absence of any commercial or financial relationships that could be construed as a potential conflict of interest.
